# Purif ied Protein Fraction of Garlic Extract Modulates Cellular Immune Response against Breast Transplanted Tumors in BALB/c Mice Model

**Published:** 2013-05-05

**Authors:** Marzieh Ebrahimi, Zuhair Mohammad Hassan, Ali Mostafaie, Narges Zare Mehrjardi, Tooba Ghazanfari

**Affiliations:** 1 Department of Immunology, School of Medical Sciences, Tarbiat Modares University, Tehran, Iran; 2 Department of Regenerative Biomedicine at Cell Science Research Center, Royan Institute for Stem Cell Biology and Technology, ACECR, Tehran, Iran; 3 Medical Biology Research Center, Kermanshah University of Medical Sciences, Kermanshah, Iran; 4 Immunoregulation Research Center, Shahed University, Tehran, Iran

**Keywords:** Garlic (Allium sativum), Protein, Tumor Infiltrating Lymphocytes (TIL), Immunomodulation, Breast cancer

## Abstract

**Objective:**

Garlic (Allium sativum) has anti-inflammatory, anti-mutagenesis, and immunomodulatory properties that modulate anti-tumor immunity and inhibit tumor growth. In this study we have examined the effect of a protein fraction isolated from fresh garlic on anti-tumor response and intra-tumor lymphocyte infiltration.

**Materials and Methods:**

In this experimental study a protein fraction was purified from fresh garlic bulbs using ultra-filtration, followed by chromatofocusing, and SDS-PAGE analysis. Anti-tumor activity was assessed by intra-tumor injection of the protein fraction and garlic extract, itself, into groups of 5 mice each. The percentage of peripheral blood and intra-tumor CD4^+^ and CD8^+^ cells were assessed by flow cytometry. Unpaired student’s t test using the SPSS program was applied for all statistical analyses.

**Results:**

Garlic extract included different type of proteins with different molecular weight. One of protein’s fraction was immunomodeulator and was composed of three single polypeptides, with molecular masses of ~10-13 kDa and different isoelectric points (pI). These molecules augmented the delayed type hypersensitivity (DTH) response compared to the control group. Intra-tumor injection of the fraction provoked a significant increase in the CD8^+^ subpopulation of T-lymphocytes, as well as a decrease in tumor size. The fraction increased peripheral blood CD8^+^ T-lymphocytes in treated animals.

**Conclusion:**

The data confirms that protein fractions purified from fresh garlic bulbs augment CD8^+^ T-cell infiltration into the tumor site, inhibiting tumor growth more efficiently than garlic extract. These ﬁndings provide a basis for further investigations on the purified polypeptide as a useful candidate for immunomodulation and tumor treatment.

## Introduction

Garlic (Allium sativum), a member of the Lily family, has been widely used as an ancient folk medicine in India, Egypt, Greece, Rome, and China for a variety of sicknesses, including: abdominal pain, parasitic infections, insect and snake bites, hemorrhoids, and rheumatism (1).

Garlic has also been thought to posses numerous other therapeutic activities such as anti-atherosclerosis (2), anti-carcinogenesis (3), anti-mutagenesis (4) and antibiotic activities (5, 6). It has been shown to inhibit the growth of transplantable tumors and reduce the incidence of certain spontaneously occurring tumors (7). There is also evidence for the immunomodulatory potential of garlic (8) or selected garlic components, which show an increased T-lymphocyte blastogenesis, natural killer (NK) activity, phagocytosis (9), modulation of cytokine production in Leishmania major-infected BALB/c mice, and changing the outcome of the immune response toward Th1 (IFN-λ, IL-2) (10).

Although tumor cells attempt to escape immune surveillance, T-lymphocytes play an important role in the host defense against tumors. Observations show that the presence of T-helper (CD4^+^) and cytolytic (CD8^+^) cells are required for tumor rejection *in vitro*. The fact that T-cell levels are reduced during disease progression *in vivo* implies that these cells contribute to tumor rejection (11). Lymphocytes that migrate into the tumor site (tumor infiltrating lymphocytes or TILs) are useful for immunotherapy because they represent an enriched population of cells that have specific reactivity to the autologus tumor (11). Changes in the sub-population of T-lymphocytes, as well as the CD4/CD8 ratio in TLIs by tumor immunotherapy may provide an important tool for evaluating the outcome of immunomodulative agents and a basis for future improvements of immunotherapy (12, 13).

In the present study we sought to determine if a protein fraction of fresh garlic can induce intratumor infiltration of lymphocytes *in vivo* and if the purified protein fraction affects tumor size. We purified a 10-13 KDa protein fraction from garlic bulbs, which was injected intra-tumor following delayed type hypersensitivity (DTH) assay and compared it to garlic extract. The results showed that the fraction augmented DTH to sheep red blood cells (sRBCs) and increased T-cell infiltration, particularly T CD8^+^, both inside the tumor and in peripheral blood.

## Materials and Methods

### Animals

Female inbred BALB/c mice (8 to 10 weeks old) were purchased from Pasteur Institute, Tehran, Iran. The animals were given sterilized water and autoclaved standard mouse chow throughout the study.

### Garlic extraction

Fresh garlic bulbs were obtained from a local market in Hamadan Province in Western Iran. The extract was prepared according to the method described by Mantis (14). Briefly, garlic bulbs were peeled and homogenized with one part distilled water in a blender. The homogenized blend was filtered under a vacuum through Whatman paper and the filtrate centrifuged at 5000 rpm for 30 minutes. The clear supernatant was sterilized through a 0.22 µ Millipore filter and stored refrigerated. Garlic extract was then run through an Amicon ultra filter system using the following membranes: 300 xm, 100 xm, 50 xm, 30 pm, and 10 pm. The fractions were collected as residue (R): R100, R50, R30, and R10 and filtrate (F), F10. Finally, the fractions were analyzed using SDS-PAGE.

### Chromatofocusing

The chromatofocusing method was used to separate proteins according to their isoelectric point (PIs). The R10 fraction was dialyzed overnight against 25 mM imidazole at a pH of 7.4 and loaded onto a chromatofocusing column (10×150 mm) that was packed with polybuffer exchangers 94 (PBE94; Amersham Pharmacia Biotec) pre-equilibrated with the same buffer (15). After sample loading, polybuffer 74 that had been diluted 12 times with distilled water and adjusted to pH=4 with HCl was passed through the column to elute the proteins according to their pIs. The flow rate of the elution polybuffer was 45 ml/h. The fractions were collected and their pH was assayed by a laboratory pH meter and absorbance read at 280 nm by a spectrophotometer.

### SDS-PAGE analysis

A 15% (w/v) polyacrylamide gel was used to determine the purity and molecular mass of the isolated molecules, as previously described by Laemmeli (16), and provided an estimate of the molecular mass with standard proteins (Pharmacia). After electrophoresis, the gel was fixed with 20% trichloro acetic acid (TCA) for 30 minutes and stained with Coomassie brilliant blue G250.

### Lymphocyte proliferation assay

The 3-(4, 5-dimethylthiazol-2-yl)-2, 5-diphenyltetrazolium bromide (MTT) assay was used to determine the effect of garlic fractions on lymphocyte proliferation.

Mouse splenocytes were used for the lymphocyte proliferation assay. Briefly, spleen tissues were aseptically collected from mice, cut into small pieces, and cells were isolated from the tissues by gently using a sterile needle. The upper portion of the mixture that contained the splenocytes was transferred to a fresh centrifuge tube and centrifuged at 1000 x g for 5 minutes, followed by the addition of 1 ml of erythrocyte lysis buffer to the pellet. Finally, lymphocytes were washed twice with culture medium and resuspended in 1 ml of RPMI 1640 that contained 10% FCS. For the MTT assay, 104 lymphocytes with different dosages of garlic fractions (0.01-0.04 mg/ml) were added to at least 100 µl per well in a 96-well plate and incubated in 5% CO_2_ at 37˚C for 24 hours. Phytohemagglutinin (PHA, 10 µg/ml) was used as the positive control and the medium culture was used as the negative control.

Next, 10 µl MTT (5 mg/ml in PBS) was added and the culture incubated for 4 hours at 37˚C in an atmosphere of 5% CO_2_ in the dark. In metabolically active cells MTT was reduced to insoluble, dark purple formazan crystals, which were dissolved in 100 µl isopropanol/HCl. Their absorbance was measured at 570 nm by a UV-visible spectrophotometer microplate reader (VersaMax, Molecular Device, USA). The stimulation index (SI) was calculated as follows: SI=540 nm absorbance in the test group/540 nm absorbance in the negative control.

For each group, experiments were repeated in triplicate and measurements were also taken in triplicate.

### Delayed type hypersensitivity

The DTH response was evaluated by priming the mice with 1×10^8^ sRBC injected subcutaneously in the back of each mouse on day 0 (5 mice in each group). The sensitized animals were challenged with 1×10^8^ sRBC injected subcutaneously on the left hind footpad on day 5. The increase in footpad thickness was measured after 24, 48 and 72 hours using a Mauser dial caliper (Germany) and the results were expressed as the percentage of increase in footpad thickness (17).

### Tumor transplant and evaluation

A single cell suspension that contained 2×10^6^ spontaneous mammary tumor cells was injected subcutaneously into 20 female BALB/c mice. Tumor incidence was evaluated by daily inspection and palpation. Tumor volume was measured by a Vernier caliper and calculated as follows: V=1/2 x L(W) 2 (18), in which V=volume, L=length, and W=width.

### Intra-tumor evolution of T-cell subpopulation

Three groups of tumorized mice (n=5 per group) were selected. The first and second groups received daily inoculation of either 20 mg/kg of R10 or garlic extract into the lesion up to 7 days. The third group received daily inoculation of saline, intra-lesion. After 7 days, the animals were killed and the solid tumors were removed, then cut into two or more small pieces with a forceps and scalpel. The pieces were rinsed twice in phosphate buffered saline (pH=7.2), then mechanically cut into very small pieces in RPMI1640 (Sigma) and 10% FCS. The suspension was passed through a 150 micron stainless steel mesh. Cells were washed twice with RPMI1640 and labeled with monoclonal antibodies.

### Immunophenotyping of T-cells in peripheral blood and tumor

We applied dual color staining with monocolonal antibody against mouse CD4 and CD8 for peripheral blood and intra-tumor lymphocytes. We established the reference immunophenotypic pattern using standard procedures. Briefly, 100 µl (1×10^6^ cells) each of the blood and tumor cells were treated as follows: each sample was immunostained with 10 µl mAbs that were directly conjugated with fluorescein isothiocyanate (FITC) or R-phycoerythrin (RPE) in a Q-Prep apparatus. Afterwards, three immunopreps were added: 0.7 ml immunoprep A (formic acid, 1.2 ml/L), 0.32 ml immunoprep B (sodium carbonate 6.0 g/L; sodium chloride 14.5 g/L; sodium sulfate 31.3 g/L), and 0.14 ml immunoprep C (paraformaldehyde 10.0 g/L, phosphate buffer 9 coulter). Each sample was then kept at 2-8˚C, in the dark for ~24 hours. Cell samples were measured on a Coulter flow cytometer with a serial filter configuration. The analysis was focused on the lymphoid areas of the forward and side scatters. Double stained cells were analyzed using Coulter software.

### Statistical analysis

To determine the statistical significance for our data, we used the un-paired student’s t test. In all analyses, statistical significance was p<0.05.

## Results

### Purification of proteins from garlic extract

**Fig 1:**
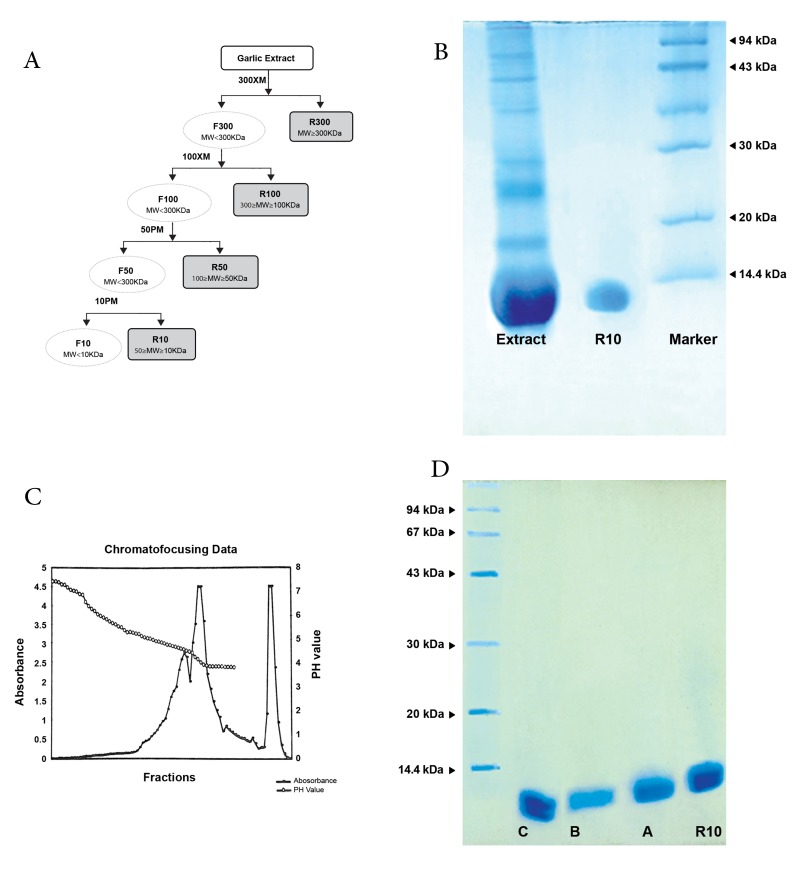
A. Schematic diagram of R10 purification by ultra-filtration; B. SDS-Page electrophoresis of partial purified garlic extract by Amicon ultra-filtration; C. Chromatofocusing graph of R10 fraction; D. SDS-Page electrophoresis of R10 fraction purified by chromatofocusing (R10A, B, C).

As shown in figure 1A, four different types of proteins were purified from fresh garlic extract according to their molecular mass using Amicon Ultrafiltration. These fractions were R300, R100, R50, and R10. Each fraction had the following pH values: 6.68 (garlic extract), 6.99 (R300), 7.31 (R100), 7.3 (R50), and 7.41 (R10). In SDS-PAGE, the R10 fraction showed one band at about 13 kDa (Fig 1B), which included three types of proteins (R10A, R10B, and R10C) with different isoelctric points as purified by chromate focusing (Fig 1C). The R10A fraction with 12.5 KDa molecular mass was eluted at pH=4.54, the R10B with the same molecular mass was eluted at pH=4.13, and the R10C included two different bands with 11.5 and 12.5 KDa that were highly negative and eluted after the application of polybuffer 74 that contained 0.1 M of NaCl (Fig 1C, D).

### Effect of garlic protein fraction on lymphocyte proliferation and delayed type hypersensitivity response

**Fig 2:**
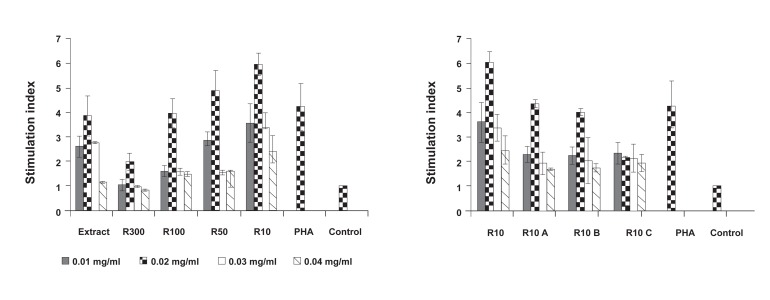
Lymphocyte proliferation in presence of different doses of garlic fractions; A. protein fractions purified by ultra-filtration, B. sub-fractions of R10 by chromatofocusing. Each test was performed in triplicate. Data presented as mean ± SD. The significance was calculated at p≤0.05.

In order to assess the effect of isolated protein fractions of garlic extract on lymphocyte proliferation, an MTT assay was performed after the spleen lymphocytes were treated with different fraction concentrations. As shown in figure 2A, 0.02 mg/ml of all fractions significantly induced lymphocyte proliferation (p≤0.03). However, R300 and R10C fractions did not stimulate lymphocyte proliferation when compared to PHA as a positive control (p≥0.05, Fig 2A, B). Of all the fractions, R10 significantly increased T-cell proliferation, followed by garlic extract, R100, R50, R10A, and R10B, which increased T-cell proliferation at approximately ≥4-fold versus the negative control. Finally, R300 and R10C increased mouse lymphocyte proliferation approximately 2-fold versus the negative control, similar to PHA (Fig 2A, B).

Cellular immune response was evaluated by the DTH assay. We divided 45 mice into 9 groups for this assay (Table 1). All injections were performed at a dose of 20 mg/kg. As shown in table 1, R10 treated animals caused a significant increase in DTH responses within 24, 48, and 72 hours. When compared to the control group (p=0.001), the optimum response was observed at 24 hours. Among the different fractions, garlic extract, R10, R10A, and R10B enhanced the DTH response (p<0.001). R10 was more effective than R10A and R10B at 24 hours. R300 and R100 decreased the DTH response (p<0.01), but R50 and R10C did not significantly induce DTH compared to the control group (p>0.05).

The data suggested that the 10 kDa fraction of garlic extract promoted the most efficient cellular response

**Table 1:** The effect of garlic fractions on the delayed type hypersensitivity response in mice


Dose	Increase in foot pad	Primer sequence Mean ± SEM (%) ** 24 hours	48 hours	Spleen histology *** 72 hours

**Group 1 sRBC + garlic extract thickness **	18.73 ± 0.53	11.14 ± 0.33	8.84 ± 0.66	Enlarged follicles
**Group 2 sRBC + R300 follicle**	8.44 ± 0.41*	6.67 ± 0.63	5.37 ± 0.55	Necrosis, atrophy of
**Group 3 sRBC+100**	11.57 ± 0.46*	9.46 ± 0.38	6.75 ± 0.82	Normal follicle picture
**Group 4 sRBC + R50**	17.25 ± 0.75	14.05 ± 0.74	10.89 ± 0.41	Normal picture,very low response
**Group 5 sRBC + R10**	46.84 ± 1.24*	18.34 ± 0.66	15.94 ± 0.53	Enlarged follicle thicknes
**Group 6 sRBC + R10A**	35.59 ± 0.89*	19.71 ± 0.68	14.35 ± 0.56	ND
**Group 7 sRBC + R10B**	24.39 ± 1.14*	17.33 ± 0.64	10.97 ± 0.83	ND
**Group 8 sRBC + R10C**	18.9 ± 0.46	13.41 ± 0.76	9.49 ± 0.93	ND
**Group 9 sRBC + saline**	16.17 ± 0.66	9.26 ± 0.48	6.17 ± 0.67	Normal picture

*P<0.05; Significant difference compared with saline group according to the t test , SEM; standard error of mean , ** ; Mean price % of footpad increase in delayed type hypersensitivity (DTH), *** ; Spleen stained with hematoxylin and eosin and ND; Not don.

### Modulation of intra-tumor lymphocyte by R10 fraction of fresh garlic

**Fig 3:**
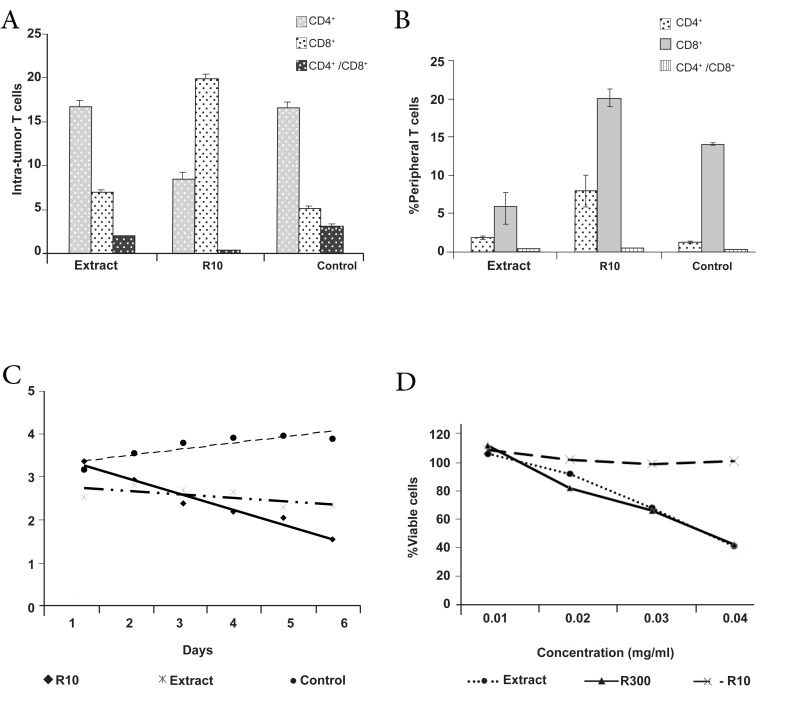
A. The effect of R10 fraction of garlic on the infiltration of T-cells; B. Peripheral T-cells; C. Tumor growth; D. Cytotoxic effect on tumor cells. Each test in A and B was performed in five replicates and triplicates in C and D. Data were presented as mean ± SD (A, B) or Mean (C, D), and the significance calculated as p≤0.05.

The injection of 2×10^6^ cells per mouse showed that among 20 injected mice, 18 developed tumors (incidence rate: 90%), which might be related to the high concentration of infused tumor cells. To determine the effect of the R10 fraction on tumor growth intra tumor- infiltrated T-lymphocyte, 15 mice with tumors were divided into 3 groups and inoculated with 20 mg/kg of either the R10 fraction, garlic extract, or PBS, daily for 7 days. As shown in figure 3, the R10 fraction increased the CD8^+^ subpopulation of intratumor T-lymphocytes (23.4 ± 1.26%) compared to 5 ± 0.97% in the control group (p<0.01, Fig 3A). An increase was noted in CD8 + peripheral blood cells (1.7 ± 0.12% in the R10 group versus 4.56 ± 1.06% in the control group, p<0.05, Fig 3B), which was concomitant with a reduction in tumor size (Fig 3C).

We sought to determine whether the reduction in tumor size was dependent on the increase in intra-tumoral cellular immunity, or if the fractions and garlic extract directly killed the tumor cells by performing a cytotoxity test. The results revealed that the number of cells decreased about 2 times in non purified garlic extract however, the number of cells in R10 group was constant before and after treatment with R10 fraction at 0.02 mg/ml.

## Discussion

Garlic is a remarkable medicinal herb with broad therapeutic properties, ranging from antibacterial to anti-cancer and anti-coagulant effects. Garlic has been shown to enhance various immune factors such as the phagocytic (cell killing) activity of macrophages, T-lymphocyte activity, killer cell activity, and antibody production (10). It has been reported that organosulfides (19), fructans (20), lectins (21), and Sallyl- L-cysteine sulfoxide (22) in fresh and old garlic are responsible for immunomodulatory potential. However, few studies (23-25) have addressed the effect of garlic proteins on the immune system.

In this study we purified a protein fraction of garlic (<13 KDa) that could augment a DTH response at a dose of 20 mg/kg in mice, which confirmed our previous data (24). Histological studies showed hyperplasia and hypertrophy of the periateriolar lymphoid sheath of the spleen and paracortical zone of lymph nodes following R10 injection in mice (9). Next, we focused more on the R10 fraction and its immunmodulatory activity. Further purification of R10 by chromatofocusing resulted in three protein subfractions (R10A, R10B, R10C) that had dif - ferent pI and nearly similar molecular masses (ranging from 11-13 kDa) were isolated. Interestingly, similar to the R10 fraction, R10A and R10B significantly (p<0.005) enhanced DTH response, whereas R10C did not exhibit any increase in the DTH response compared with the controls. Wen et al (26) have reported the existence of two major proteins that constitute approximately 96% of total garlic proteins, of weights 14 kDa and 40 kDa. As we have reported in this study, Chandrashekar and Venkatesh (23) also purified three different molecules in the range of 13 KDa in aged garlic and determined their immunomodulatory and mannose binding activities.

In general, it has been shown that garlic inhibits tumor growth both *in vitro* and *in vivo* (27-29) however the exact mechanism of its inhibition is unclear. It seems that an increase of intra-tumor infiltration of the lymphocyte and activation of T-cells (29) inhibits tumor growth *in vivo*. In the current study, intra-tumor inoculation of only the R10 fraction and its subfractions significantly increased infiltration of the CD8^+^ lymphocyte subpopulation, shift in CD4/CD8 ratio and decrease of tumor volume. A decrease in tumor size was unrelated to the cytotoxity of the R10 fraction or its subfractions. Ghazanfari et al. (10) also indicated that R10 fractions caused a shift to Th1 cytokines in Leshmania infected mice. According to Clement et al. (21) they have proposed that garlic’s immunomodulatory activity of the two subfractions purified from the 30 KDa ultrafiltrate of raw garlic were markedly similar to the abundant Allium sativum agglutinins (ASA) I and II. In a similar study, Morioka and colleagues (30) purified a protein of around 13 KDa (F4) from aged garlic. They reported that F4 enhanced the cytotoxicity of human peripheral blood lymphocytes (PBL) against both natural-killer (NK)-sensitive K562 and NK-resistant M14 cell lines. F4 actually enhanced IL-2-induced proliferation and IL-2 receptor (Tac) expression of PBL, without a significant increase in IL-2 production.

## Conclusion

We determined that although garlic extract reduced tumor growth by increasing CD8 Tcell infiltration into the tumor site and caused cytotoxic effects, the R10 purified fraction was more efficient than garlic extract on modulating anti-tumor immune response without causing considerable cytotoxity on tumor cells. Finally, R10 and its subfractions might be considered as potential candidates for cell mediated therapy and *in vivo* applications.

## References

[1] Butt MS, Sultan MT, Butt MS, Iqbal J (2009). Garlic: nature’s
protection against physiological threats. Crit
Rev Food Sci Nutr.

[2] Budoff MJ, Ahmadi N, Gul KM, Liu ST, Flores FR, Tiano J (2009). Aged garlic extract supplemented
with B vitamins, folic acid and L-arginine retards
the progression of subclinical atherosclerosis: a
randomized clinical trial. Prev Med.

[3] Tsubura A, Lai YC, Kuwata M, Uehara N, Yoshizawa K (2011). Anticancer effects of garlic and garlicderived
compounds for breast cancer control. Anticancer
Agents Med Chem.

[4] Jeong HG, Lee YW (1998). Protective effects of diallyl
sulfide on N-nitrosodimethylamine-induced immunosuppression
in mice. Cancer Lett.

[5] Focke M, Feld A, Lichtenthaler K (1990). Allicin, a naturally
occurring antibiotic from garlic, specifically
inhibits acetyl-CoA synthetase. FEBS Lett.

[6] Tedeschi P, Maietti A, Boggian M, Vecchiati G, Brandolini V (2007). Fungitoxicity of lyophilized and
spray-dried garlic extracts. J Environ Sci Health B.

[7] Lau BH, Tadi PP, Tosk JM (1990). Allium Sativum (garlic)
and cancer prevention. Nutr Res.

[8] Kyo E (2001). Immunomodulatory effects of aged garlic
extract. J Nutr.

[9] Ishikawa H, Saeki T, Otani T, Suzuki T, Shimozuma K, Nishino H (2006). Aged garlic extract prevents
a decline of NK cell number and activity in patients
with advanced cancer. J Nutr.

[10] Ghazanfari T, Hassan ZM, Ebtekar M, Ahmadiani A, Naderi G, Azar A (2000). Garlic induces a shift
in cytokine pattern in Leishmania major-infected
BALB/c mice. Scand J Immunol.

[11] Hilders CG, Ras L, van Eendenburg JD, Nooyen Y, Fleuren GJ (1994). Isolation and characterization of
tumor-infiltrating lymphocytes from cervical carcinoma. Int J Cancer.

[12] Hernberg M (1999). Lymphocyte subsets as prognostic
markers for cancer patients receiving immunomodulative
therapy. Med Oncol.

[13] Estrela-Lima A, Araújo MS, Costa-Neto JM, Teixeira-
Carvalho A, Barrouin-Melo SM, Cardoso SV (2010). Immunophenotypic features of tumor infiltrating
lymphocytes from mammary carcinomas in female
dogs associated with prognostic factors and survival
rates. BMC Cancer.

[14] Mantis AJ (1979). Effect of Garlic extract on food poisoning
bacteria. Lebensm Wiss U Technol.

[15] Mantle TJ, Noone P (1992). Choromatofocusing. In: Doonan
S, editor. Methods in molecular biology, protein
purificsation protocols.

[16] Laemmli UK (1970). Cleavage of structural proteins during
the assembly of the head of bacteriophage T4. Nature.

[17] Ebtekar M, Hassan ZM (1993). Effect of immunomodulators
pyrimethamine and cimetidine on immunosuppression
induced by sulfur mustard in mice. Int J
Immunopharmacol.

[18] Singh SV, Mohan RR, Agarwal R, Benson PJ, Hu X, Rudy MA (1996). Novel anti-carcinogenic activity
of an organosulfide from garlic: inhibition of H-RAS
oncogene transformed tumor growth *in vivo* by diallyl
disulfide is associated with inhibition of p21Hras
processing. Biochem Biophys Res Commun.

[19] Wilasrusmee C, Siddiqui J, Bruch D, Wilasrusmee S, Kittur S, Kittur DS (2002). *in vitro* immunomodulatory
effects of herbal products. Am Surg.

[20] Chandrashekar PM, Prashanth KV, Venkatesh YP (2011). Isolation, structural elucidation and immunomodulatory
activity of fructans from aged garlic extract. Phytochemistry.

[21] Clement F, Pramod SN, Venkatesh YP (2010). Identity of
the immunomodulatory proteins from garlic (Allium
sativum) with the major garlic lectins or agglutinins. Int Immunopharmacol.

[22] Hui C, Like W, Yan F, Tian X, Qiuyan W, Lifeng H (2010). S-allyl-L-cysteine sulfoxide inhibits tumor necrosis
factor-alpha induced monocyte adhesion and intercellular
cell adhesion molecule-1 expression in
human umbilical vein endothelial cells. Anat Rec
(Hoboken).

[23] Chandrashekar PM, Venkatesh YP (2009). Identification
of the protein components displaying immunomodulatory
activity in aged garlic extract. J Ethnopharmacol.

[24] Ghazanfari T, Hassan ZM, Ebrahimi M (2002). Immunomodulatory
activity of a protein isolated from
garlic extract on delayed type hypersensitivity. Int
Immunopharmacol.

[25] Hassan ZM, Yaraee R, Zare N, Ghazanfari T, Sarraf
Nejad AH, Nazori B (2003). Immunomodulatory affect
of R10 fraction of garlic extract on natural killer activity. Int Immunopharmacol.

[26] Wen GY, Mato A, Wisniewski HM, Malik MN, Jenkins EC, Sheikh AM (1995). Light and electron microscopic
immunocytochemical localization of two
major proteins in garlic bulb. J Cell Biochem.

[27] Gao CM, Takezaki T, Ding JH, Li MS, Tajima K (1999). Protective effect of allium vegetables against both
esophageal and stomach cancer: a simultaneous
case-referent study of a high-epidemic area in Jiangsu
Province, China. Jpn J Cancer Res.

[28] Sundaram SG, Milner JA (1996). Diallyl disulfide suppresses
the growth of human colon tumor cell xenografts
in athymic nude mice. J Nutr.

[29] Lau BH, Yamasaki T, Gridley DS (1991). Garlic compounds
modulate macrophage and T-lymphocyte
functions. Mol Biother.

[30] Morioka N, Sze LL, Morton DL, Irie RF (1993). A protein
fraction from aged garlic extract enhances cytotoxicity
and proliferation of human lymphocytes mediated
by interleukin-2 and concanavalin A. Cancer
Immunol Immunother.

